# Assessment of Satisfaction Rate After Closed Reductions of Nasal Bone Fractures: A Cross-Sectional Study

**DOI:** 10.7759/cureus.80662

**Published:** 2025-03-16

**Authors:** Ahmad Alharthi, Lina Y Natto, Johara A Alnafie, Ahmed H Althqafi, Alaa Babkour

**Affiliations:** 1 Otolaryngology - Head and Neck Surgery, Alhada Armed Forces Hospital, Taif, SAU; 2 Otolaryngology - Head and Neck Surgery, King Abdullah Medical City, Makkah, SAU; 3 Otolaryngology, Al-Noor Specialist Hospital, Makkah, SAU

**Keywords:** closed reduction, facial cosmetic surgery, nasal bone fracture, nasal obstruction, nasal obstruction symptom evaluation (nose) scale, nasal trauma, saudi arabia

## Abstract

Background: The nose is the central and most protruded part of the face, most vulnerable to facial trauma. Treatment for nasal bone fractures involves open reduction and closed reduction. Closed nasal reduction is a simple procedure that can be performed under local or general anesthesia with an acceptable success rate.

Aim: The current study aimed to assess the patient's satisfaction following the closed reduction of nasal bone fractures, both in aesthetic and functional values, and causes of dissatisfaction.

Methodology: A community-based descriptive cross-sectional study was conducted in Al-Noor Specialist Hospital, Makkah, Saudi Arabia, targeting all patients who underwent close reduction of nasal bone fracture between December 2021 and December 2022. The severity of the nasal obstruction was assessed using the validated Nasal Obstruction Symptom Evaluation (NOSE) scale, and selected questions from the Glasgow Benefit Inventory (GBI) were used to assess the change in general health status. The final questionnaire was distributed to the eligible patients through Google Forms (Google LLC, Mountain View, California, United States).

Results: A total of 55 patients with reduced nasal bone fractures were included. Patients' ages ranged from 20 to 55 years with a mean age of 33.1±12.9 years. Precisely 39 (70.9%) patients were male. A total of 17 (30.9%) nasal injuries were due to accidents, and 15 (27.3%) were due to slips/falls. After closed reduction surgery, 42 (76.4%) were satisfied with their nose cosmesis, but nine (16.3%) were unsatisfied. For unsatisfied cases after surgery, the main reason was their own perception (61.5%), opinion of relatives/friends (30.8%), or medical opinion (7.7%). A significant relation between postoperative complaints and the severity of nasal symptoms was reported (P < 0.05).

Conclusions: The study revealed a high satisfaction rate for the closed reduction method, mainly in married older patients. Clinical complaints, severity of symptoms, and associated health changes decreased after undergoing closed reduction.

## Introduction

The nose serves as a vital sensory and respiratory organ, playing a pivotal role in the body's defense mechanisms against external threats, both through physical barriers and immunological responses [1]. Moreover, it constitutes a key element of facial aesthetics, significantly influencing speech production and ensuring the integrated appearance of other facial features such as the lips, forehead, cheeks, and eyebrows [2,3]. Positioned centrally on the face and proximal to significant facial tissues, its delicate and fragile structure makes it susceptible to a variety of traumas [4]. Nasal fractures, defined as breaks or disruptions in the bone structure of the nose, are the most prevalent type of facial fracture and rank third among the most common fractures within the skeletal system [5,6].

Nasal fractures predominantly affect men, who are approximately twice as likely to experience nasal fractures as women, with the highest frequency occurring during the second and third decades of life [7,8]. Typically, fractures involve the upper portion of the nasal pyramid, with injuries to the central facial region also resulting in fractures to this bone [9]. The leading causes of nasal fractures include physical activities, falls, and assaults. The rise in various physical injuries, including nasal trauma, can be attributed to technological advancements, increased use of motor vehicles, escalating instances of conflict and violence, as well as the prevalence of sports-related accidents, occupational accidents, and falls, among other factors [3].

Closed reduction is generally reserved for simple, non-comminuted nasal fractures, although there are exceptions to this protocol [10]. However, performing closed reduction without direct visualization can lead to several complications, including insufficient correction, the creation of new fractures, mucosal damage, and nasal hemorrhage [11]. The technique of closed reduction offers numerous benefits such as its simplicity, safety, ease of application, and minimal morbidity risk [12]. Given that achieving an "ideal" outcome for the patient is unlikely in closed reduction, the primary goal is to minimize deformity and functional impairments. This study aims to evaluate patient satisfaction following closed reduction of nasal bone fractures (NBFs) in Makkah, Saudi Arabia. The primary outcomes assessed using a validated scale include functional improvement, aesthetic satisfaction, and overall quality of life. Additionally, the study seeks to identify key factors influencing these outcomes.

## Materials and methods

This was a community-based descriptive cross-sectional study conducted at Al-Noor Specialized Hospital, Makkah, targeting all patients who underwent closed reduction of NBFs between December 2021 and December 2022. The Institutional Review Board, Ministry of Health, Makkah Al-Mukarramah Region, approved the study (approval number: H-02-K-076-0622-752). Individuals under 18 years of age and those who declined participation or did not complete the study survey were excluded. 

Data collection

Data collection from Al-Noor Hospital's medical records encompassed medical history, injury mechanisms, patient complaints, and the surgical procedures performed. 

Study Tool

An online questionnaire (see Appendices) was used to gather information on participants' demographics, socioeconomic status, educational level, post-operative clinical assessments, complaints, and satisfaction levels. The severity of nasal obstruction was evaluated using the validated Nasal Obstruction Symptom Evaluation (NOSE) scale [13], and select items from the Glasgow Benefit Inventory (GBI) [14,15] were employed to assess changes in general health status. 

The questionnaire was initially developed in English and subsequently translated into Arabic to ensure accessibility for all participants. The translation process followed a forward and backward translation method, where independent bilingual experts translated the questionnaire into Arabic, followed by a reverse translation to English to ensure accuracy and conceptual equivalence. An initial assessment of the questionnaire's validity and reliability was conducted with a sample of 10 participants, resulting in a Cronbach's alpha coefficient of 0.71. The development phase of the questionnaire involved a thorough literature review and expert consultations, leading to its subsequent validation and the creation of its definitive version. In an effort to ensure broad accessibility, the finalized questionnaire was distributed to the intended audience through a Google Forms link (Google LLC, Mountain View, California, United States), remaining open for responses until saturation was reached with input from eligible respondents.

Surgical procedure

Closed reduction was performed under general anesthesia. The procedure involved the use of Asch forceps and Walsham forceps for fracture realignment. Post-reduction, splints were used to maintain alignment.

Statistical analysis

After the initial extraction, the data were thoroughly reviewed and coded, then subsequently entered into IBM SPSS Statistics for Windows, Version 22.0 (Released 2013; IBM Corp., Armonk, New York, United States). Statistical analyses were performed utilizing two-tailed tests, with a p-value < 0.05 indicating statistical significance. A descriptive analysis encompassing frequency and percentage distributions was conducted for all variables. This included the sociodemographic data of the study participants, their field of employment, income levels, smoking status, and monthly income. Furthermore, details regarding nasal bone injuries such as the mechanisms of injury, patient complaints, and the methods used for repair were systematically organized in tabular form. Additionally, graphical representations were employed to illustrate the medical assessments conducted before and after surgery, as well as any changes in health status. The frequency of patient satisfaction with their nasal appearance post surgery was also examined. Cross-tabulation was utilized to explore the relationship between postoperative patient satisfaction and variables such as demographic data and clinical assessments, applying Pearson's chi-square test and the Monte Carlo test for analyses involving small frequency distributions.

## Results

A total of 55 cases eligible for reduced NBF were included (Table 1). The age range of the patients was 20-55 years, with a mean age of 33.1 ± 12.9 years. Of these, 39 (70.9%) were male, 52 (94.5%) were of Saudi nationality, 28 (50.9%) were married, and 25 (45.5%) were single. In terms of education, 22 (40%) had completed secondary education, and 19 (34.5%) had attained a university-level education or higher. A monthly income of less than 5,000 Saudi riyals (SR) was reported in 29 (52.7%) cases, whereas 20 (36.4%) had a monthly income ranging from 5,000 to 15,000 SR. Additionally, 22 (40%) participants were smokers, and 49 (89.1%) resided in urban areas. Regarding employment, 33 (60%) were unemployed, 20 (36.4%) worked outside the healthcare field, and only two were employed within the healthcare sector.

**Table 1:** Sociodemographic data of study participants (N=55)

Variables	Frequency	Percentage
Age (years)		
20-30	19	34.5%
30-40	21	38.2%
40+	15	27.3%
Gender		
Male	39	70.9%
Female	16	29.1%
Marital Status		
Single	25	45.5%
Married	28	50.9%
Divorced	2	3.6%
Educational Level		
Below secondary	14	25.5%
Secondary	22	40.0%
University and above	19	34.5%
Monthly Income (in Saudi Riyal (SR))		
< 5000	29	52.7%
5000-15000	20	36.4%
> 15000	6	10.9%
Nationality		
Saudi	52	94.5%
Non-Saudi	3	5.5%
Smoking		
Yes	22	40.0%
No	33	60.0%
Residence		
Urban	49	89.1%
Rural	6	10.9%
Employment Field		
Unemployed	33	60.0%
Non-Medical field	12	21.8%
Military field	8	14.5%
Medical field	2	3.6%

Table 2 presents a comprehensive elaboration on the nature of NBFs, mechanisms of injury, patient complaints, and methods of repair among the study participants. Accidents were the cause of 17 (30.9%) nasal injuries, slips or falls accounted for 15 (27.3%), beatings for 14 (25.5%), and sports activities for nine (16.4%). Associated injuries comprised maxillofacial fractures in 12 (21.8%), brain injuries in two (3.6%), and other types of injuries in another two (3.6%) patients. The primary complaints prior to surgery were nasal obstruction and nasal deformity (each reported by 19 (34.5%) patients), followed by epistaxis in 11 (20%). Regarding the timing for undergoing closed reduction, 28 (50.9%) patients waited for 5-10 days, 18 (32.7%) waited for less than five days, and nine (16.4%) waited for more than 10 days. The majority of cases (n=50, 90.9%) underwent a closed reduction in the operating room (OR), while five (9.1%) required a combination of reduction and other procedures.

**Table 2:** Nasal bone injury, mechanism, complaints and repair methods among study participants (N=55)

Nasal injury data	Frequency	Percentage
Mechanism of injury		
Accidents	17	30.9%
Slip\fall	15	27.3%
Beating	14	25.5%
Sports	9	16.4%
Associated injury or fracture		
None	39	70.9%
Maxillofacial fractures	12	21.8%
Brain injury	2	3.6%
Others	2	3.6%
Complaint before intervention		
None	4	7.3%
Nasal obstruction	19	34.5%
Nasal deformity	19	34.5%
Epistaxis	11	20.0%
Hyposmia	2	3.6%
Time between nasal bone fracture and reduction		
< 5 days	18	32.7%
5-10 days	28	50.9%
10-15 days	5	9.1%
> 15 days	4	7.3%
Methods of reduction		
Closed reduction in OR	50	90.9%
Reduction with other procedures	5	9.1%

**Figure 1:**
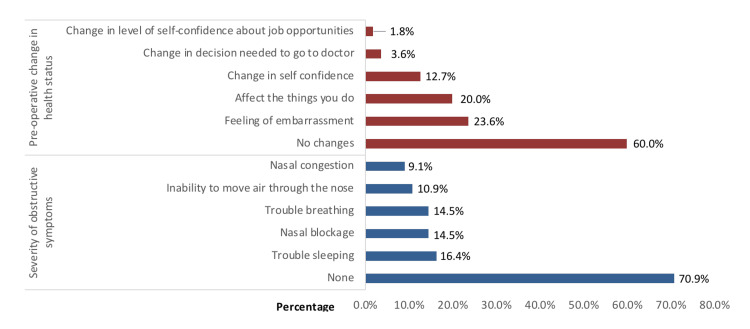
Results of preoperative medical assessment (severity and health assessment) in study partiicpants (N=55)

In the medical evaluation detailed in Figure 1, a significant number of patients with NBFs reported varying degrees of symptom severity and health status before surgery. A substantial portion of the cases (n=39, 70.9%) exhibited no severe symptoms. Among the reported symptoms, difficulties sleeping were reported by nine (16.4%), nasal blockage by eight (14.5%), breathing difficulties by eight (14.5%), challenges breathing through the nose by six (10.9%), and nasal congestion by five (9.1%). Additionally, 33 (60%) participants reported no changes in health, while 13 (23.6%) felt embarrassment, 11 (20%) observed that their daily activities were impacted, and seven (12.7%) noted changes in self-confidence.

**Figure 2:**
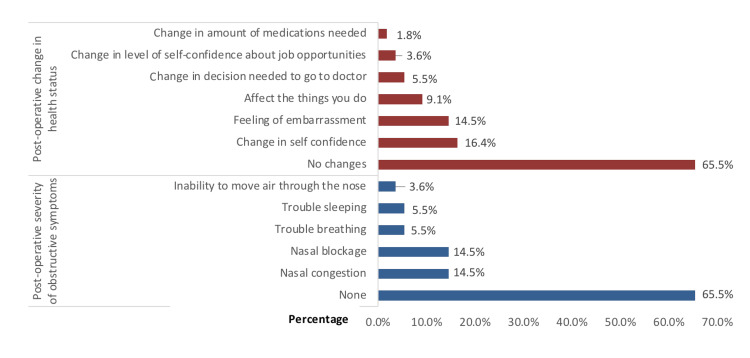
Results of postoperative medical assessment (severity and health assessment) in study participants (N=55)

The assessment after surgery in Figure 2 showed that 36 (65.5%) patients did not experience severe nasal symptoms. Nasal congestion and blockage were noted by eight (14.5%) patients each, and three (5.5%) reported issues with breathing or sleeping. In terms of postoperative health changes, 36 (65.5%) indicated no changes, an increase in self-confidence was seen in nine (16.4%), feelings of embarrassment were experienced by eight (14.5%), and five (9.1%) felt their daily activities were affected. Most study participants (n=38, 69.1%) experienced no postoperative complaints, whereas nasal obstruction and deformity were each reported by seven (12.7%) patients, epistaxis by two (3.6%), and a solitary case involved hyposmia.

In addition, a comparison of patient satisfaction levels before and after the closed reduction of NBFs is detailed in Table 3. Initially, 37 (67.3%) participants expressed satisfaction with their nasal appearance, whereas 14 (25.5%) were dissatisfied. Post procedure, the satisfaction rate increased to 42 (76.4%), albeit nine (16.3%) did not share this positive outcome. Among those dissatisfied postoperatively, the primary reasons included personal perception (n=8, 61.5%), feedback from relatives or friends (n=4, 30.8%), and medical evaluations (n=1, 7.7%).

**Table 3:** Preoperative versus postoperative satisfaction regarding closed reductions of nasal bone fracture in study participants (N=55)

Satisfaction	Frequency	Percentage
Preoperative satisfaction rate with cosmesis		
Very dissatisfied	5	9.1%
Dissatisfied	9	16.4%
Undecided	4	7.3%
Satisfied	5	9.1%
Very satisfied	32	58.2%
Postoperative satisfaction rate with cosmesis		
Very dissatisfied	2	3.6%
Dissatisfied	7	12.7%
Undecided	4	7.3%
Satisfied	9	16.4%
Very satisfied	33	60.0%
If not satisfied, your dissatisfaction was driven by		
Your own view regarding the result post surgery	8	61.5%
Opinion of relatives or friends	4	30.8%
Medical opinion	1	7.7%

Furthermore, an examination of the demographic factors that impact satisfaction reveals a noteworthy correlation (Table 4). Patients aged 40 and above universally reported satisfaction, contrasting with only 61.9% (n=13) showing in the younger age group of 20-30 years, marking a statistically significant difference (p = .049). Marital status also played a pivotal role, with 25 (89.3%) married individuals satisfied post surgery compared to 17 (63%) unmarried individuals (p = .031). However, analyses revealed that other demographic variables were not significantly associated with levels of satisfaction, as indicated by p-values greater than 0.05 across all remaining categories.

**Table 4:** Relation between satisfaction and demographic data for patients undergone closed reductions of nasal bone fracture P: Exact probability test;  * P < 0.05 (significant)

Sociodemographics	Postoperative satisfaction rate with cosmesis						p-value
	Dissatisfied		Undecided		Satisfied		
	Frequency	Percentage	Frequency	Percentage	Frequency	Percentage	
Age (years)							0.049*
10-20	4	21.1%	1	5.3%	14	73.7%	
20-30	5	23.8%	3	14.3%	13	61.9%	
40+	0	0.0%	0	0.0%	15	100.0%	
Gender							0.405
Male	8	20.5%	3	7.7%	28	71.8%	
Female	1	6.3%	1	6.3%	14	87.5%	
Marital Status							0.031*
Unmarried	8	29.6%	2	7.4%	17	63.0%	
Married	1	3.6%	2	7.1%	25	89.3%	
Educational Level							0.464
Below secondary	1	7.1%	1	7.1%	12	85.7%	
Secondary	6	27.3%	1	4.5%	15	68.2%	
University and above	2	10.5%	2	10.5%	15	78.9%	
Monthly Income (in Saudi Riyal (SR))							0.333
< 5000	7	24.1%	3	10.3%	19	65.5%	
5000-15000	2	10.0%	1	5.0%	17	85.0%	
> 15000	0	0.0%	0	0.0%	6	100.0%	
Nationality							0.661
Saudi	8	15.4%	4	7.7%	40	76.9%	
Non-Saudi	1	33.3%	0	0.0%	2	66.7%	
Residence							0.766
Urban	8	16.3%	4	8.2%	37	75.5%	
Rural	1	16.7%	0	0.0%	5	83.3%	
Employment							0.474
Unemployed	7	21.2%	2	6.1%	24	72.7%	
Employed	2	9.1%	2	9.1%	18	81.8%	
Smoking							0.186
Yes	6	27.3%	1	4.5%	15	68.2%	
No	3	9.1%	3	9.1%	27	81.8%	

Following the demographic analysis, Table 5 presents the relationship between patient satisfaction and clinical assessments after closed reductions of NBFs. Notably, patients exhibiting postoperative challenges, such as the inability to breathe through the nose, did not report satisfaction, in stark contrast to those with nasal blockage (50% satisfaction) and patients exhibiting no postoperative nasal symptoms (88.9% satisfaction), indicating a significant difference (p = .001). Similarly, satisfaction was absent among patients experiencing postoperative hyposmia, as opposed to 14.3% (n=1) expressing satisfaction in those with nasal deformities, and a notable satisfaction rate (n=32, 88.9%) among those with no postoperative obstruction symptoms (p = .001). Furthermore, a higher satisfaction rate (n=30, 83.3%) was observed in patients without any postoperative health changes, compared to those experiencing such changes (n=12, 63.2%) (p = .049).

**Table 5:** Relation between satisfaction clinical assessment of patients undergone closed reductions of nasal bone fracture P: Exact probability test;  * P < 0.05 (significant)

Clinical assessment	Post-operative satisfaction rate with cosmesis						p-value
	Dissatisfied		Undecided		Satisfied		
	Frequency	Percentage	Frequency	Percentage	Frequency	Percentage	
Preoperative severity of obstructive symptoms							0.189
None	6	15.4%	1	2.6%	32	82.1%	
Nasal congestion	1	20.0%	0	0.0%	4	80.0%	
Nasal blockage	2	25.0%	0	0.0%	6	75.0%	
Trouble breathing	1	12.5%	2	25.0%	5	62.5%	
Trouble sleeping	1	11.1%	2	22.2%	6	66.7%	
Inability to breathe through the nose	2	33.3%	0	0.0%	4	66.7%	
Complaint before intervention							0.145
None	0	0.0%	0	0.0%	4	100.0%	
Nasal obstruction	2	10.5%	2	10.5%	15	78.9%	
Epistaxis	1	9.1%	0	0.0%	10	90.9%	
Nasal deformity	6	31.6%	1	5.3%	12	63.2%	
Hyposmia	0	0.0%	1	50.0%	1	50.0%	
Preoperative change in health status							0.859
Yes	4	18.2%	2	9.1%	16	72.7%	
No	5	15.2%	2	6.1%	26	78.8%	
Postoperative severity of obstructive symptoms							0.001*
None	1	2.8%	3	8.3%	32	88.9%	
Nasal congestion	3	37.5%	0	0.0%	5	62.5%	
Nasal blockage	3	37.5%	1	12.5%	4	50.0%	
Trouble breathing	1	33.3%	0	0.0%	2	66.7%	
Trouble sleeping	1	33.3%	0	0.0%	2	66.7%	
Inability to move air through the nose	2	100.0%	0	0.0%	0	0.0%	
Postoperative complaint							0.001*
None	0	0.0%	2	5.3%	36	94.7%	
Nasal obstruction	4	57.1%	0	0.0%	3	42.9%	
Epistaxis	0	0.0%	0	0.0%	2	100.0%	
Nasal deformity	5	71.4%	1	14.3%	1	14.3%	
Hyposmia	0	0.0%	1	100.0%	0	0.0%	
Postoperative change in health status							0.049*
Yes	6	31.6%	1	5.3%	12	63.2%	
No	3	8.3%	3	8.3%	30	83.3%	

## Discussion

Within the spectrum of maxillofacial injuries, NBFs stand out for their prevalence, accounting for 39% of fractures in this category [16,17]. These injuries occur more frequently in men than in women [16]. Given the prevalence of such injuries, conducting epidemiological studies and providing optimal care are imperative.

This study was conducted to evaluate patient satisfaction following closed NBF reduction in Makkah City. The findings indicate that the majority of cases involved male patients under 40 years of age, a demographic characterized by high levels of physical activity. Moreover, most patients resided in urban settings, suggesting that a dynamic lifestyle and the increased likelihood of trauma, including facial trauma, are associated with city living. Accidents, falls, and assaults were identified as the primary mechanisms of injury for NBFs, with approximately one-third of the cases involving concomitant injuries, predominantly maxillofacial fractures resulting from accidents.

Supporting these observations, Kaladagi et al. in their study had reported an average patient age of 40.61 years (SD ± 15.83), with a distribution of 36.21% female and 63.7% male patients [18]. They found road traffic accidents (RTAs) to be the leading cause of NBFs, accounting for 55.17% of cases. Additionally, other studies have indicated a higher incidence of these fractures among males, predominantly attributed to trauma [19,20]. This discrepancy may stem from men being more inclined to participate in manual labor and outdoor activities compared to women [21]. Sadhoo et al. found a significant proportion of nasal fractures (68.5%) occurring in urban areas, attributed to the dense population and higher RTA rates, followed by falls and assaults [22]. In India, Sinha et al. noted that violence was responsible for 29.35% of NBFs, with RTAs themselves constituting 35.3% of the causes [19]. Renkonen et al. also highlighted violence as the primary cause, representing 43.3% of cases [23]. Moreover, patients within the intermediate age groups reported a higher prevalence of NBFs [19,20], aligning with the patterns observed in the findings of the present study.

In terms of symptom severity, the majority of cases presented with mild symptoms, although some individuals experienced difficulties sleeping, nasal blockage, and issues with breathing, including the inability to move air through the nose. Regarding health status prior to surgery, fewer than two-thirds of the patients reported no changes in their health, while approximately one-fourth experienced feelings of embarrassment, and one-fifth noted an impact on their daily activities. The postoperative assessment did not reveal any significant changes in symptom severity or overall health status. The most commonly reported preoperative complaints included nasal obstruction, nasal deformity, and epistaxis. These observations align with findings from Al Arfaj et al., who noted that nasal deformity (96%), depressed fracture (42.2%), and nasal obstruction resulting from trauma (20.4%) were the predominant clinical presentations [24]. Additional clinical symptoms such as pain, epistaxis, swelling over the nasal dorsum, further nasal deformities, and wounds have also been documented, often presenting simultaneously [25-27].

In evaluating patient satisfaction following closed reduction, the present study revealed that over three-fourths of participants expressed satisfaction, with fewer than 10% indicating a neutral level of satisfaction. This marks an increase from pre-surgical levels, where approximately two-thirds were satisfied with their nasal appearance. Notably, satisfaction rates were higher among older and married individuals, as well as those who did not report postoperative symptoms, health changes, or complaints.

In contrast, Al Arfaj et al. [24] reported a significantly lower satisfaction rate of 44.2% following closed reduction, while Wild et al. [28] observed an 80% satisfaction rate, closely aligning with the findings of this study. Similarly, satisfaction rates of 71% were documented by Watson et al. [29] and 65% by Yilmaz et al. [30] among adults, with a slightly lower rate of 62% in children. Furthermore, Hung et al. noted that 71% of patients were content with the outcome of closed reduction, though approximately one-third would consider additional surgery to address residual nasal deformity [31]. Kaladagi et al. found that 60.3% of patients were pleased with the functional results of closed reduction for NBF, whereas 41.38% expressed satisfaction with the aesthetic results [18]. Robinson et al. highlighted a high satisfaction level, with 95% of patients content with both functional and aesthetic postoperative outcomes [15]. Likewise, Abu-Samra et al. reported a 75% satisfaction rate following closed reduction [32], corroborating the high satisfaction rates associated with this conservative treatment approach, as concluded in the current study.

The current study revealed that over half of the participants delayed their visit to the emergency room for management by 5-10 days, whereas approximately one-third sought care within the first five days. The optimal timing for employing closed reduction remains ambiguous and varies according to different sources [33,34]. It is advised that the reduction be performed as promptly as possible, although the presence of edema may necessitate a brief waiting period before proceeding with the reduction.

Study limitations

This study has limitations that need to be acknowledged. The follow-up period may not have been long enough to capture longer-term complications or dissatisfaction that may arise after the closed reduction of NBFs. Additionally, the reliance on Google Forms for survey distribution may have excluded individuals with limited internet access or digital literacy.

## Conclusions

This investigation highlighted that the majority of nasal fractures occurred in middle-aged males residing in urban settings, with the predominant cause being accidents, often accompanied by other types of fractures, especially maxillofacial fractures. The most commonly reported clinical symptoms included deformity and obstruction, which persisted in some individuals even after undergoing reduction. In terms of patient satisfaction, the findings indicate a high level of satisfaction with the closed reduction technique, particularly among married, older individuals, who did not experience postoperative complaints or health changes. Thus, the results emphasize the critical need for prompt and adept management of nasal fractures to maximize patient outcomes and satisfaction levels.

## Appendices

Questionnaire

**Table 6:** Study Questionnaire

Section	Question number	Question	Response options
Socio-demographical Data	1	Marital status	Married, Single, Divorced
	2	Age	10 - 60 years (Open-ended)
	3	Gender	Male, Female
	4	Level of education	Illiterate, Elementary, Intermediate, High school, Bachelor’s and above
	5	Socioeconomic status	Low (<5000 SR), Medium (5000-15000 SR), High (>15000 SR)
	6	Nationality	Saudi, Non-Saudi
	7	Smoking	Yes (details: duration, type, packs/day), No, Former (when quit?)
	8	Residence	Urban, Rural
	9	Occupation	General education, Medical, Commercial, Military, Unemployed
Clinical Data	10	Time between nasal bone fracture and reduction	<5 days, 5-10 days, 10-15 days, >15 days
	11	Mechanism of injury	Beating, Accidents, Sports, Slip/Fall, Other
	12	Associated injury/fracture	Maxillofacial fractures, Brain injury, Other
	13	Methods of reduction	Closed reduction in OR, Reduction with other procedure
	14	Complaints before intervention	Nasal obstruction, Epistaxis, Nasal deformity, Hyposmia, Other
	15	Satisfaction rate with cosmesis preoperative	Very satisfied (80-100%), Satisfied (60-79%), Undecided (50-59%), Dissatisfied (40-49%), Very dissatisfied (<40%)
	16	Satisfaction rate with cosmesis postoperative	As above
	17	Any postoperative complaint	Nasal obstruction, Epistaxis, Nasal deformity, Hyposmia, Other
Severity of Obstructive Symptoms	18	Preoperative Score	Nasal congestion, Blockage, Trouble breathing, Trouble sleeping, Inability to move air
	19	Postoperative Score	Same as above
Health Status Changes	20	Preoperative changes	Affected daily activities, Embarrassment in group, Self-confidence, Time needed for doctor visits, Job opportunities, Self-consciousness, Medication needs
	21	Postoperative Changes	Same as above
	22	If unsatisfied, reason?	Self-view of surgery result, Opinion of relative/friend, Medical opinion
